# Atypical Lower Extremity Discoid Lupus Erythematosus Associated With Newly Diagnosed Systemic Lupus Erythematosus in a Male Patient: A Case Report

**DOI:** 10.7759/cureus.110629

**Published:** 2026-06-10

**Authors:** Shamas Rafique

**Affiliations:** 1 Internal Medicine, BayCare Medical PC, New York, USA; 2 Internal Medicine, First Affiliated Hospital of Xinjiang Medical University, Urumqi, CHN; 3 Medicine and Surgery, Xinjiang Medical University, Urumqi, CHN; 4 Medicine and Surgery, BeeWell International Hospital, Islamabad, PAK

**Keywords:** anti-dsdna antibodies, atypical presentation, cutaneous lupus, discoid lupus erythematosus, hydroxychloroquine, hypocomplementemia, lower extremity lesions, male lupus, systemic lupus erythematosus

## Abstract

Discoid lupus erythematosus (DLE) classically affects sun‑exposed areas, such as the face, scalp, and ears. Involvement of the lower extremities is uncommon, and presentation of DLE in association with systemic lupus erythematosus (SLE) in a male patient is relatively rare. This case report describes a 34‑year‑old man who presented with chronic, scaly, dyspigmented plaques confined to both the lower legs, which prompted further evaluation and led to the diagnosis of SLE. Histopathological evaluation supported the diagnosis of DLE, while serologic workup revealed high‑titer antinuclear antibodies (ANAs), anti‑double‑stranded DNA (anti‑dsDNA), anti‑Smith antibodies, and hypocomplementemia, supporting a diagnosis of SLE with chronic cutaneous lupus involvement. Following treatment with strict photoprotection, topical corticosteroids, and weight‑based hydroxychloroquine, the patient showed marked clinical and serologic improvement at the 12‑week and 9‑month follow‑ups. This case highlights the importance of evaluating for systemic involvement in patients with biopsy‑supported DLE, particularly when lesions occur in atypical anatomical locations and in populations traditionally considered at lower risk.

## Introduction

Discoid lupus erythematosus (DLE) is the most common form of chronic cutaneous LE and occurs in a subset of patients with systemic lupus erythematosus (SLE), although it may also exist as an isolated cutaneous entity [1,2]. SLE demonstrates a marked female predominance, with a female‑to‑male ratio of approximately 9:1, contributing to potential diagnostic delays in men [3]. Biopsy‑supported DLE confined predominantly to the lower extremities as the presenting manifestation leading to the diagnosis of previously unrecognized SLE in a young male represents an uncommon clinical scenario, underscoring the need for heightened clinical awareness.

DLE typically involves photo‑exposed regions, particularly the face, scalp, and ears [4]. Lesions arising in relatively sun‑protected areas, such as the lower extremities, are uncommon and may obscure early recognition. Lower leg plaques are frequently misdiagnosed as vascular conditions (e.g., stasis dermatitis) or common inflammatory and fungal disorders (e.g., psoriasis or tinea), thereby delaying appropriate systemic workup. Furthermore, while cutaneous lupus may precede systemic disease, progression from localized DLE to SLE remains clinically variable.

This case is presented to emphasize that SLE should remain an important diagnostic consideration in patients with atypical DLE distribution, regardless of sex, particularly when accompanied by subtle systemic features. The report further illustrates that lower extremity‑predominant DLE, even in the absence of classic photosensitive or malar manifestations, can be the clinical finding that uncovers previously undiagnosed systemic autoimmunity.

## Case presentation

A 34‑year‑old previously healthy man (Fitzpatrick skin type IV) presented with a six‑month history of progressively enlarging, non‑pruritic, non‑painful lesions over both lower legs. The lesions began as small erythematous macules and gradually evolved into well‑demarcated plaques with adherent scale, follicular plugging, and dyspigmentation. He worked as an office administrator and reported no significant outdoor occupational or recreational sun exposure; he did not use tanning beds and typically wore long trousers. He was a lifelong non‑smoker and denied occupational friction, chronic heat exposure, repetitive trauma, or athletic activities involving the lower extremities. There was no medication history suggestive of drug‑induced lupus, and he had not tried any over‑the‑counter topical treatments. There was no known family history of autoimmune diseases, including SLE, rheumatoid arthritis, or psoriasis. Informed consent was obtained from the patient for publication of this case report and accompanying images.

He denied preceding trauma, insect bites, or topical exposures. There was no history of photosensitivity, malar rash, oral ulcers, alopecia, or Raynaud's phenomenon. However, he reported intermittent fatigue and mild morning stiffness involving the knees and wrists lasting less than 30 minutes. He denied chest pain, dyspnea, neurologic symptoms, or constitutional features. Vital signs were normal (blood pressure 118/72 mmHg, heart rate 76 bpm, respiratory rate 16/min, temperature 36.8°C). Body mass index was 24 kg/m². No peripheral edema or lymphadenopathy was present.

**Figure 1:**
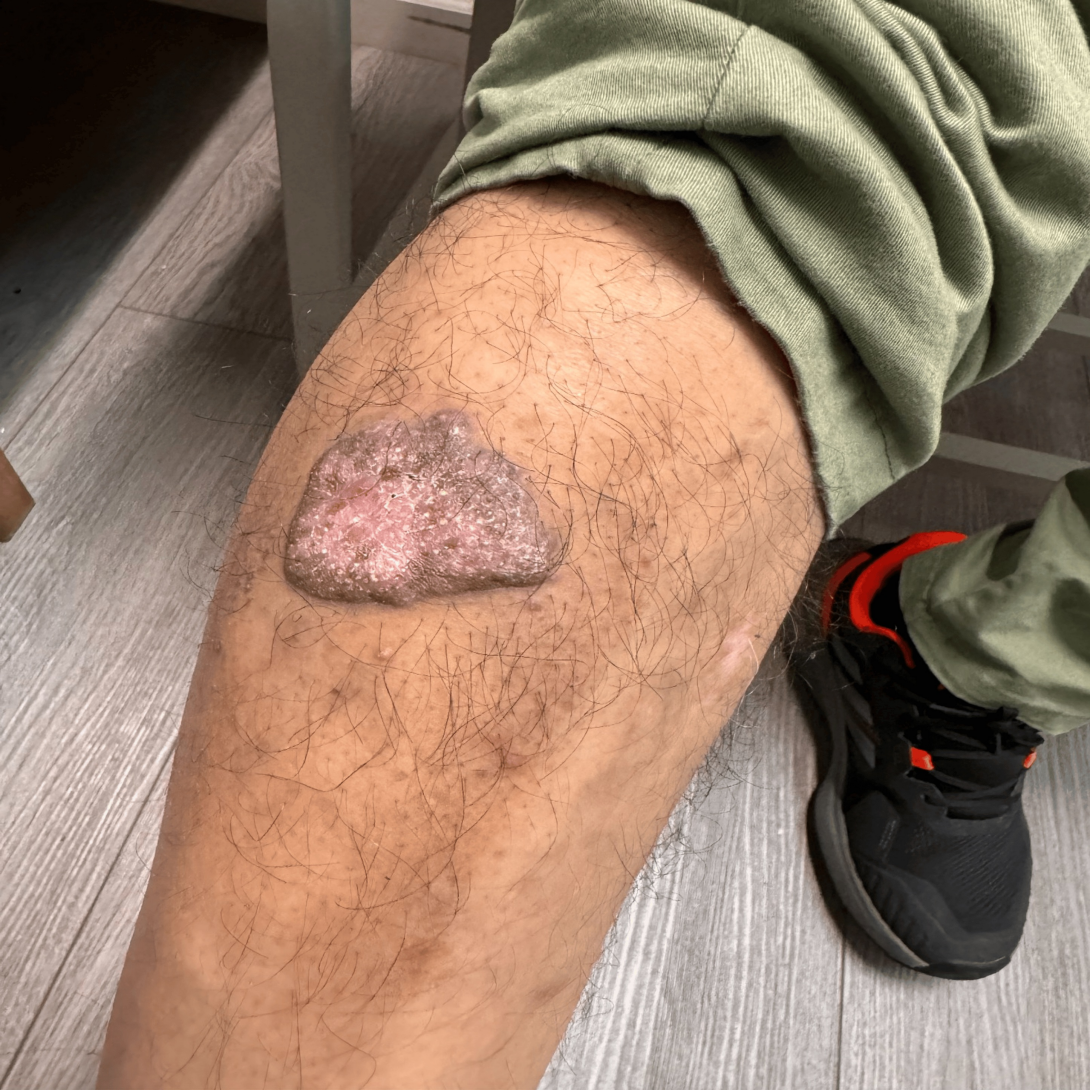
Discoid lupus erythematosus involving the right lower extremity A well‑demarcated hyperkeratotic discoid plaque over the anterior right lower leg demonstrated adherent scale, follicular plugging, central atrophic hypopigmentation, and peripheral post‑inflammatory hyperpigmentation without ulceration.

Examination of the scalp, nails, oral mucosa, and nasal mucosa revealed no discoid lesions or ulcerations. Musculoskeletal examination demonstrated a full range of motion without joint swelling or tenderness. On the right lower leg, a large, well‑demarcated discoid plaque was noted over the anterior aspect (Figure 1). The lesion measured approximately 5‑6 cm in diameter and exhibited a hyperkeratotic surface with adherent scale and prominent follicular plugging. The central portion appeared hypopigmented and atrophic with a pinkish hue, while the periphery showed irregular hyperpigmentation and mild induration. The surface texture was rough and scaly, consistent with chronic cutaneous lupus. There was no clinical evidence of venous insufficiency, varicose veins, or stasis dermatitis. No ulceration or secondary infection was observed. Similar but smaller lesions were present on the contralateral leg; only the right lower extremity lesion is shown, as it was the larger and more representative lesion. No lesions were identified on the face, scalp, ears, or trunk. There was no synovitis, mucosal ulceration, or evidence of serositis. Neurological and cardiopulmonary examinations were unremarkable. The diagnosis was discussed with dermatology and rheumatology consultants, who agreed with the assessment.

Investigations

Punch biopsy from an active lesion demonstrated epidermal atrophy with compact hyperkeratosis and prominent follicular plugging. At the dermoepidermal junction, vacuolar interface dermatitis with basal keratinocyte degeneration was identified. The dermis showed superficial and deep perivascular and periadnexal lymphocytic infiltrates extending around adnexal structures, together with increased dermal mucin deposition. Periodic acid‑Schiff staining highlighted basement membrane zone thickening. Fungal stains were negative for dermatophytes. These findings were considered characteristic of DLE and correlated closely with the clinical presentation [5]. The absence of neutrophilic microabscesses, regular psoriasiform epidermal hyperplasia, granulomatous inflammation, or fungal elements helped exclude important clinical mimics, including psoriasis, necrobiosis lipoidica, and dermatophytosis. Histopathologic images were unavailable because original digital files were not retained; therefore, independent visual review by readers is not possible. Nevertheless, the pathology report was reviewed in detail and described clinicopathologic features characteristic of DLE. Direct immunofluorescence was not performed; however, it is recognized as a useful adjunct in the diagnosis of cutaneous lupus. The combination of characteristic clinicopathologic findings and highly specific serologic abnormalities strongly supported the diagnosis of DLE in the appropriate clinical context, and additional testing was not expected to alter management.

Laboratory findings are summarized in Table 1. ANA was detected by indirect immunofluorescence on HEp‑2 cells at a titer of 1:1280 with a speckled pattern. Anti‑dsDNA antibodies were measured by enzyme‑linked immunosorbent assay (ELISA) and were >60 IU/mL (laboratory upper reporting limit). Hemoglobin (14.2 g/dL), platelet count (210 × 10⁹/L), and total leukocyte count (5.8 × 10⁹/L) were within normal limits, with isolated mild lymphopenia (1.0 × 10⁹/L) noted. Urinalysis was unremarkable, with no proteinuria, hematuria, or casts. Serum creatinine (0.9 mg/dL) and blood urea nitrogen (14 mg/dL) were normal, and a spot urine protein‑to‑creatinine ratio was <0.5 g/g (laboratory-reported value), confirming the absence of renal involvement.

**Table 1:** Baseline laboratory findings *Value exceeded the laboratory upper reporting limit. ANA: antinuclear antibody, anti‑dsDNA: anti‑double‑stranded DNA, IU/mL: international units per milliliter, Sm: Smith, C3: complement component 3, C4: complement component 4, mg/dL: milligrams per deciliter.

Parameter	Patient value	Reference range
ANA (titer, pattern)	1:1280, speckled	<1:80
Anti‑dsDNA (IU/mL)	>60*	<30
Anti‑Smith (Sm)	Positive	Negative
Complement C3 (mg/dL)	55	90-180
Complement C4 (mg/dL)	8	10-40
Lymphocyte count (×10⁹/L)	1.0	1.5-4.0
Anti‑Ro/SSA, anti‑La/SSB	Negative	Negative

Differential diagnosis

The differential diagnosis included psoriasis, lichen planus, tinea corporis, necrobiosis lipoidica, stasis dermatitis, hypertrophic lupus erythematosus, lichen simplex chronicus, cutaneous sarcoidosis, and mycosis fungoides. Psoriasis was excluded by the absence of neutrophilic microabscesses on histopathology and lack of Auspitz's sign. Lichen planus typically presents with violaceous, pruritic, polygonal papules and histologically shows a band‑like lymphocytic infiltrate with wedge‑shaped hypergranulosis; these features were absent. Tinea corporis was ruled out by negative fungal stains. Necrobiosis lipoidica was inconsistent with the absence of necrobiotic collagen and palisading granulomas. Stasis dermatitis was unlikely, given the lack of pitting edema, varicosities, hemosiderin deposition, and venous stasis changes on clinical examination. Hypertrophic lupus erythematosus and hypertrophic lichen planus were considered but were less consistent with the atrophic center and histologic interface pattern without marked psoriasiform epidermal hyperplasia. Lichen simplex chronicus was excluded because the lesion was not pruritic and lacked the characteristic lichenification with epidermal hyperplasia. Cutaneous sarcoidosis was excluded by the absence of granulomas on biopsy. Mycosis fungoides was unlikely given the stable, non-infiltrated plaque and the absence of atypical lymphocytes or epidermotropism on histopathology. Potassium hydroxide preparation and bacterial culture were not performed because fungal stains were negative, and there was no clinical evidence of secondary infection. Dermoscopy was not available at the time of evaluation. Venous insufficiency was excluded by clinical examination. The combination of characteristic DLE histopathologic findings and supportive serology strongly supported the diagnosis.

Diagnosis and management

The patient fulfilled the 2019 EULAR/ACR classification criteria for SLE with a cumulative score of 14, consisting of chronic cutaneous lupus (4 points), low C3 and C4 (4 points), and SLE‑specific antibodies (anti‑dsDNA or anti‑Sm; 6 points), in the setting of a positive ANA at a titer of ≥1:80 [1]. Notably, isolated lymphopenia is not part of the 2019 classification criteria (which award points only for leukopenia <4.0 × 10⁹/L), so it did not contribute to the score. Nevertheless, the patient met the threshold of ≥10 points, fulfilling the classification criteria for SLE. The clinical diagnosis of SLE was made by the treating team based on the compatible cutaneous disease, positive ANA entry criterion, SLE‑specific antibodies, hypocomplementemia, and the exclusion of alternative explanations. Mild lymphopenia was considered an additional, albeit non‑scoring, hematologic feature.

The patient was initiated on strict photoprotection, including daily use of broad‑spectrum SPF 50+ sunscreen, and was counseled on the importance of adherence even for lower extremity lesions [6]. Topical clobetasol propionate 0.05% ointment was prescribed twice daily for localized lesions with a plan to taper after clinical improvement and transition to a lower‑potency topical corticosteroid [7]. Intralesional corticosteroids were considered but not required, given the response to topical therapy. Systemic therapy with hydroxychloroquine (HCQ) 200 mg twice daily was initiated in accordance with current SLE management guidelines [8,9]. The patient weighed 82 kg, resulting in a daily HCQ dose of approximately 4.9 mg/kg of actual body weight, which is within the recommended ≤5 mg/kg/day limit to minimize the risk of retinal toxicity [10]. The patient was counseled on the importance of lifelong smoking avoidance and regular ophthalmologic screening for hydroxychloroquine retinopathy. Baseline ophthalmologic evaluation was normal. Given the presence of anti‑dsDNA positivity and hypocomplementemia, ongoing surveillance for potential renal involvement was emphasized despite the absence of baseline renal disease; monitoring included complete blood count, complement levels, anti‑dsDNA titers, and urinalysis every three months.

Outcome and follow-up

At 12 weeks, the patient demonstrated marked improvement, with reduced erythema, scaling, and induration and residual post‑inflammatory dyspigmentation. The dyspigmentation was predominantly post‑inflammatory hyperpigmentation, a common concern in individuals with darker skin phototypes, rather than true leukoderma. Fatigue and joint stiffness resolved. Follow‑up laboratory parameters demonstrated improvement in serologic activity markers and normalization of complement levels and lymphocyte count after treatment (Table 2). These serologic changes are considered supportive of a favorable response, though a causal link to hydroxychloroquine cannot be definitively established in a single case.

**Table 2:** Follow-up laboratory findings Anti‑dsDNA: anti‑double‑stranded DNA, IU/mL: international units per milliliter, C3: complement component 3, C4: complement component 4, mg/dL: milligrams per deciliter.

Parameter	Week 0	Week 12	Reference range
Anti‑dsDNA (IU/mL)	>60	25	<30
Complement C3 (mg/dL)	55	98	90-180
Complement C4 (mg/dL)	8	18	10-40
Lymphocyte count (×10⁹/L)	1.0	1.8	1.5-4.0

No additional systemic manifestations developed over nine months of follow‑up. A chronological summary of the patient's clinical course is provided in Table 3.

**Table 3:** Timeline of clinical course

Timepoint	Clinical event
Six months before presentation	Initial lesion onset
2-5 months before presentation	Progressive plaque enlargement
At presentation	Clinical evaluation
At diagnosis	Biopsy and serologic confirmation
At diagnosis	Initiation of treatment
12 weeks after treatment initiation	Clinical and laboratory improvement
9 months after presentation	Stable disease

Patient perspective

The patient expressed initial frustration regarding the diagnostic delay, as the lesions had been present for six months before a definitive diagnosis was reached. He reported that the lesions caused self‑consciousness due to their appearance and discomfort from scaling and tightness of the skin. Following treatment, he noted a significant improvement in both the physical appearance and symptoms, which allowed him to resume normal social and occupational activities without concern. He emphasized the importance of early recognition and expressed hope that his case might help other patients with similar atypical presentations receive a timely diagnosis.

## Discussion

This case illustrates the diagnostic value of maintaining a high index of suspicion for systemic autoimmunity in patients presenting with biopsy‑supported DLE in atypical anatomical locations. Although lower extremity involvement by DLE has been described, its occurrence as the predominant cutaneous finding leading to the diagnosis of previously unrecognized SLE in a young male patient remains an uncommon presentation that may present diagnostic challenges. The lesion in this case exhibited classic discoid morphology, hyperkeratosis, follicular plugging, central atrophy, and dyspigmentation despite occurring in a relatively photo‑protected area and without typical provoking factors. Notably, the patient lacked traditional aggravating factors for chronic cutaneous lupus, including smoking, which has been associated with increased disease severity and reduced antimalarial responsiveness [11]. Although the lower leg is relatively sun‑protected, low‑grade friction from clothing or daily activities may have contributed to lesion localization, although the precise reason for lower extremity involvement in this patient remains uncertain. Lower extremity DLE plaques may be mistaken for venous dermatitis, psoriasis, or lichen simplex chronicus, leading to delayed systemic workup and disease‑modifying therapy.

Furthermore, cutaneous lupus may serve as the presenting clinical feature that leads to recognition of previously undiagnosed SLE, even in male patients. In the present case, although the lower extremity plaques were the patient's primary complaint, concurrent serologic abnormalities and hypocomplementemia were already present at diagnosis, indicating that systemic autoimmunity was established at the time of presentation. SLE in men is often underrecognized and may be associated with more severe disease and distinct serologic profiles [12]. Several studies have reported that male patients with SLE may exhibit fewer classic cutaneous manifestations and a greater frequency of renal, cardiovascular, and hematologic involvement, which can delay diagnosis when the initial presentation is limited to atypical skin lesions. This underscores the importance of maintaining diagnostic suspicion for systemic disease even when the cutaneous phenotype deviates from the expected pattern.

High‑titer anti‑dsDNA antibodies and hypocomplementemia have been associated with increased disease activity and renal involvement in SLE cohorts; however, these markers alone do not predict future nephritis in an individual patient [5]. The presence of anti‑Sm antibodies, as observed in this case, carries high specificity for SLE and has been incorporated into classification criteria to aid in the identification of systemic disease [13]. Reported progression rates from DLE to SLE vary widely depending on patient population and disease extent, with higher rates observed in patients with generalized lesions and serologic abnormalities [14,15]. Although renal involvement was absent at baseline, the combination of male sex and serologic abnormalities justified ongoing clinical surveillance for renal involvement, although no renal disease developed during the available follow‑up period.

Emerging evidence suggests that hydroxychloroquine may improve cutaneous disease control and overall lupus outcomes. Whether early treatment prevents progression from cutaneous lupus to systemic disease remains an area of ongoing investigation [16]. Although dermoscopy was not performed, dermoscopic findings reported in DLE include follicular keratotic plugs, perifollicular whitish halos, telangiectasias, and structureless white scarring areas, which may aid in differentiating DLE from psoriasis and lichen planus in atypical presentations. Selected studies addressing DLE progression, atypical presentations, and associated systemic disease are summarized in Table 4.

**Table 4:** Literature comparison ANA: antinuclear antibody, DLE: discoid lupus erythematosus, SLE: systemic lupus erythematosus.

Study	Main finding relevant to the current case
Chong et al. [14]	Positive ANA associated with a greater likelihood of progression from DLE to SLE
Fredeau et al. [11]	Generalized cutaneous lupus and systemic features associated with increased risk of severe SLE
Alharbi et al. [2]	Progression from DLE to SLE varies substantially across studies; serologic abnormalities increase concern for systemic disease
Current case	Lower extremity‑predominant DLE led to recognition of previously undiagnosed SLE in a male patient

These findings suggest that clinicians should maintain a low threshold for autoimmune serologic testing in patients with biopsy‑supported DLE, even when lesions occur in non‑photo‑exposed regions or in demographic groups considered lower risk for SLE. Early systemic evaluation may facilitate timely diagnosis, appropriate monitoring, and prompt initiation of therapy when systemic disease is identified.

Learning points and limitations

This case reinforces several important clinical lessons: discoid lupus erythematosus may present in atypical, non‑sun‑exposed locations, and cutaneous lupus can be the presenting feature of previously undiagnosed SLE. Male patients are at risk for delayed diagnosis, yet high‑titer ANA, anti‑dsDNA positivity, and low complement levels should prompt careful evaluation for systemic involvement. Hydroxychloroquine remains a cornerstone of treatment for both cutaneous and systemic lupus manifestations, and clinicians should maintain a low threshold for serologic evaluation in any patient with biopsy‑supported DLE. The limitations of this single case report, however, must be acknowledged. The findings are not generalizable, and the absence of a control group or statistical analysis means the observed outcomes may not be reproducible. Follow‑up was limited to nine months, which may not capture late‑onset systemic manifestations or disease flares. Histopathologic images were unavailable because original digital files were not retained; therefore, independent visual review by readers is not possible, although the written pathology report and serologic correlation provided strong clinicopathologic support for the diagnosis. Baseline dermoscopic imaging was also unavailable. Antiphospholipid antibody testing was not performed because there was no clinical history suggestive of thrombotic or obstetric antiphospholipid syndrome. Despite these constraints, the detailed clinical, histopathologic, and serologic correlation offers valuable insights for clinicians encountering atypical DLE.

## Conclusions

DLE involving the lower extremities should not be presumed to represent an isolated cutaneous process without appropriate systemic evaluation. This case illustrates the heterogeneous clinical manifestations of lupus and the importance of diagnostic vigilance in atypical presentations. Early recognition and appropriate serologic evaluation facilitate timely treatment and monitoring. Clinicians should maintain a low threshold for evaluation of SLE in patients with biopsy‑supported DLE, particularly when lesions occur in atypical locations or are accompanied by serologic abnormalities.
